# 

**Published:** 1936

**Authors:** 


### CONTENTS.

Carcinoma of the Bronchus: Page

Clinical Features. By C. Bruce Perry, M.D., F.R.C.P. .. 125

Primary Carcinoma of the Lung Radiologically Considered. By Gilbert B. Bush, M.B., Ch.B., D.M.R.E. 131

Bronchoscopic Diagnosis of Carcinoma of the Lung. By Gordon Scarff, M.B., Ch.B., F.R.C.S. .. .. .. 137

The Pathological Aspect. By Arthur L. Taylor, M.D... 139

The Treatment of Carcinoma of the Lung. By Duncan Wood, F.R.C.S. .. .. .. .. .. .. .. 145

A Fatal Case of Meningitis Due to Infection of the Sphenoidal Sinus by Pfeiffer's Bacillus, with a Short Review of Methods of Treatment. By C. Elaine Field, M.B., B.S., and Herbert Rogers, M.D. .. .. .. .. .. .. .. 151

Surgical Clinics in Scandinavia. By A. Rendle Short, M.D., B.S., B.Sc., F.R.C.S. .. .. .. .. .. .. .. 157

Report of the Committee on the Scottish Health Services. By The Editor .. .. .. .. .. .. .. .. .. 163

Reviews of Books .. .. .. .. .. .. .. .. 168

Editorial Notes .. .. .. .. .. .. .. .. 175

Meetings of Societies .. .. .. .. .. .. .. 180

The Medical Library of the University of Bristol .. .. 181

Publications Received .. .. .. .. .. .. .. 184

Local Medical Notes .. .. .. .. .. .. .. 185

